# Markers of disease severity and positive family history are associated to significant risk perception in rheumatoid arthritis, while compliance with therapy is not: a cross-sectional study in 415 Mexican outpatients

**DOI:** 10.1186/s13075-021-02440-y

**Published:** 2021-02-22

**Authors:** Irazú Contreras-Yáñez, Pilar Lavielle, Patricia Clark, Virginia Pascual-Ramos

**Affiliations:** 1 Department of Immunology and Rheumatology, Instituto Nacional de Ciencias Médicas y Nutrición Salvador Zubirán, Vasco de Quiroga 15, Belisario Domínguez, Sección XVI, 14080 Mexico City, Mexico; 2 Clinical Epidemiology Unit, Centro Médico Nacional Siglo XXI, Hospital de Especialidades, Mexico City, Mexico; 3 Head of the Clinical Epidemiology Unit, Hospital Infantil de México Federico Gómez and Facultad de Medicina UNAM, Mexico City, Mexico

**Keywords:** Rheumatoid arthritis, Risk perception, Judgment biases, Latin-American patients

## Abstract

**Background:**

Assessing risk perception (RP) helps explain how rheumatoid arthritis (RA) patients integrate their ideas concerning the disease and how this understanding affects their self-care management. Compliance with treatment impacts disease-related outcomes and could be associated with RP to variable degrees and at different levels. The primary objective was to determine a potential association between RP and compliance with therapy in RA outpatients and to identify additional factors. The secondary objective was to identify factors associated with judgment bias such as unrealistic RP.

**Patients and methods:**

Between January 2018 and June 2019, 450 consecutive outpatients who received RA-related treatment were invited to a face-to-face interview to obtain socio-demographic data, RA-related information, comorbidities, and the following outcomes: adherence, persistence, and concordance with medications assessed with a questionnaire locally designed; RP with the RP questionnaire (RPQ); disease activity with the Routine Assessment of Patient Index Data-3 (RAPID-3); disability with the Health Assessment Questionnaire Disability Index (HAQ-DI); quality of life with Medical Outcomes Study Short Form-36 (SF-36) instrument; pain and overall disease with the respective visual analogue scale (VAS); and health literacy assessed with 3 questions. Significant RP was defined according to a cut-off based on the 75th percentile value of the sample in which the RPQ was validated. Unrealistic RP was defined based on the coincidence of the presence/absence of significant RP and less/more than 7 unfavorable medical criteria. Multiple logistic regression analysis was used. Patients provided written informed consent and the study received Internal Review Board approval.

**Results:**

There were 415 patients included, primarily middle-aged women with long-standing disease and moderate disease activity. Almost half of the patients were receiving corticosteroids and 15.9% intensive RA-related treatment. There were 44.1% of the patients concordant with treatment and 22.6% had significant RP. The patients’ treatment behavior was not retained in the regression analysis; meanwhile, rheumatoid nodes, surgical joint replacement, family history of RA, and higher RAPID-3 score were associated with significant RP. There were 56 patients with unrealistic RP; significant RP and more unfavorable medical criteria were associated with unrealistic RP.

**Conclusions:**

Compliance with therapy was not associated with significant RP in RA outpatients.

## Background

Current recommendations for rheumatoid arthritis (RA) patients emphasize the use of disease-modifying anti-rheumatic drugs (DMARDs) according to a treat-to-target strategy, lifestyle changes, regular visits to the rheumatologist, laboratory testing, and sometimes, additional diagnostic procedures [1, 2]. Such complex intervention may be more successfully maintained when the rheumatologist embraces a patient-centered-care model, which has additional ethical implications [3].

RA patients’ perceived risks are related to aspects of their rheumatic disease that may positively or negatively influence their self-care-related behavior [4, 5]. Risk assessment is a discipline designed to aid in the identification, characterization, and quantification of risks [6]; meanwhile, risk perception (RP) is defined as a multidimensional phenomenon that describes the individual’s judgment of the likelihood of experiencing something unpleasant [7]. The formation of RP relies on the patient’s ability to produce, understand and use numeric information, but a number of additional factors contribute to the formation of RP [8–11]. We recently developed and validated a questionnaire, the risk perception questionnaire (RPQ), to assess RP in patients with RA; significant RP was defined according to a cut-off based on the 75th percentile value of the sample in which the RPQ was validated [12].

Assessing perceived risk may help explain how RA patients integrate their ideas concerning the disease and its treatments, and how this understanding affects their self-care management. In particular, compliance with treatment impacts disease-related outcomes [13, 14] and could be associated with RP to variable degrees and at different levels; for instance, significant RP may be associated with compliance with treatment, while judgment biases such as unrealistic optimism and pessimism, in which subjects underestimate or overestimate, respectively, the likelihood of experiencing a negative event related to their disease [15–17], could additionally modulate treatment-related behavior. Assessing RP may be conceived as a cognitive intervention, and patients with significant and/or unrealistic RP may benefit the more from such intervention.

The primary objective of the study was to comprehensively approach the RP phenomenon by identifying factors associated with significant RP in our population; in particular, we hypothesized an association between significant RP and compliance with therapy. The exploratory secondary objective was to describe the characteristics of RA patients with judgment biases and their associated factors.

## Methods

### Ethical considerations

The study was performed in compliance with the Helsinki Declaration [18]. The Research Ethics Committee of the Instituto Nacional de Ciencias Médicas y Nutrición Salvador Zubirán (INCMyN-SZ) approved the study (reference number IRE-2429).

### Study design, setting, and study population

This cross-sectional study was performed between January 2018 and June 2019 at the outpatient clinic of the Department of Immunology and Rheumatology at INCMyN-SZ.

Consecutive RA patients who had indicated at least one DMARD in the past 6 months were invited to participate. RA diagnosis was based on the treating rheumatologist’s criteria; the exclusion criterion was RA patients with an overlapping syndrome (except secondary Sjögren syndrome).

### Patient assessments

Assessments were performed the same day patients visited their primary rheumatologist and the following outcome measures were obtained: patient compliance with RA-related treatment, which was assessed based on a questionnaire developed locally (the Compliance Questionnaire, CQ) [14]; RP, which was assessed with the RPQ [12]; disease activity, which was assessed with the Spanish version of the Routine Assessment of Patients Index Data-3 (RAPID-3) [19]; disability, which was assessed with the version of the Health Assessment Questionnaire Disability Index (HAQ-DI) validated for Spanish-speaking patients [20]; quality of life, which was assessed with the Spanish version of the generic Medical Outcomes Study Short Form-36 (SF-36) instrument [21]; pain and overall disease, which were assessed with a horizontal, 0–100 mm visual analogue scale (pain-VAS, and overall disease-VAS, respectively) [22]; and health literacy, which was assessed with 3 questions validated for Spanish-speaking patients [23].

Patients had face-to-face interviews to obtain socio-demographic information, disease duration, additional relevant comorbid conditions, and RA-related and RA-unrelated treatment. Relevant comorbid condition was defined as a specific diagnosis requiring ≥ 3 related medical consultations within 1 year previous to the study entry, irrespective of a treatment indication; in addition, patients taking drug(s) for a specific diagnosis (but RA) although not recorded on the charts were considered to have relevant comorbidity (Additional file 1).

Medical records were reviewed by a single trained researcher who used standardized formats for data abstraction.

### Description of the questionnaires and scales

#### The CQ (Additional file 2)

The CQ is a 22-items questionnaire that evaluates both adherence with and persistence on therapy, and patient motivations for non-concordance with therapy. Performance of the CQ has shown high sensitivity and satisfactory specificity to detect persistence on DMARDs [14].

#### The RPQ (Additional file 3)

The RPQ is composed of 27 items distributed in 5 dimensions: likelihood of developing articular and extra-articular manifestations, likelihood of developing complications and/or comorbidities and disease severity, likelihood of developing unfavorable consequences, perception of personal responsibility to prevent and develop RA-related complications, and perception of personal control over the disease. The RPQ has been found to be valid, reliable, and feasible for assessing RP in our population. The RPQ score ranges from 0 to 100 mm, where 100 indicates the highest RP [12].

#### The RAPID-3

The RAPID-3 includes 3 measures: physical function, pain, and a global patient estimate evaluation. It has an adjusted score of 0–10, with higher scores that translate into higher disease activity [19].

#### The HAD-DI

The HAQ-DI evaluates the ability to perform activities of daily living. The final score ranges from 0 to 3, with higher levels indicating more disability [20].

#### The SF-36

The 36 items of the SF-36 are distributed into 8 subscales or domains, which are scored with values from 0 to 100, and a lower score indicates poorer health. There are 2 component summary measures, the physical component, and the mental/emotional component [21].

#### Pain-VAS and overall disease-VAS

Both scales were used as recommended by the American College of Rheumatology to evaluate pain and overall disease [22]. The pain scale assessed “today” pain, instead of pain during the period 1 week prior.

#### Health literacy questions

Three self-reported health literacy questions validated for use in Spanish-speaking populations [23] were used: “How confident are you when filling out medical forms by yourself?”, “How often do you have problems learning about your medical condition because of difficulty understanding medical information?”, and “How often do you have someone, such as a family member, friend, hospital or clinic worker or caregiver, help you read hospital materials?”. Scores range from 3 to 15, with higher scores reflecting worse self-reported health literacy [23].

### Definitions

A patient was considered to be *CQ-persistent* if, in item 10 (“In the past 6 months, how often did you completely stop taking your medication?”), boxes 0 (“Never”) or 1 (“Almost never”) were filled. A patient was considered to be *CQ-adherent* when boxes 3 (“Almost always”) or 4 (“Always”) were filled for items 12 (“In the past 6 months, I took my medication exactly at the day/s indicated by my rheumatologist”), 13 (“In the past 6 months, I took my medication exactly at the day-times indicated by my rheumatologist”), and 14 (“In the past 6 months, every time I took my medication, I took the precise amount of tablets indicated by my rheumatologist”). Finally, a patient was defined to have *concordance with therapy* if he/she was both CQ adherent and CQ persistent [14].

Significant RP was defined based on the 61.7 mm cut-off, which corresponded to the 75th percentile of the RPQ score distribution, described in the original study [12]; in order to test criterion validity, it was shown that patients with significant RP had more frequently unfavorable medical criteria recorded on their charts, when compared to their counterpart; also, logistic regression models consistently showed that patients with ≥ 3 unfavorable medical criteria had an increased risk of a RPQ score ≥ 61.7 mm [12].

Four disease activity categories were defined based on the cut-offs on the 0–30 RAPID-3 scale as follows: > 12, high; 6.1–12.0, moderate; 6.0–3.1, low; and ≤ 3, near-remission [19].

Inadequate health literacy was defined when patients scored “little” or less on the “Confident with forms” question [23].

Unrealistic RP was defined based on the coincidence of the presence/absence of significant RP and less/equal or more than 7 medical criteria (out of 10), which corresponded to the mean (5) + 1 SD (1.6) of the medical criteria distribution in the population. We repeated analysis with less/equal or more than 6 medical criteria. The 10 criteria were considered unfavorable outcomes or predictive of unfavorable outcomes and were as follows: female sex, medium-low socioeconomic status, rheumatoid factor (RF) and/or antibodies to cyclic citrullinated peptides (ACCP)-positive status, rheumatoid nodes, HAQ score ≥ 2 [24], RAPID-3 score > 12 [19], intensive treatment (≥ 3 DMARDs including corticosteroids), presence of at least one comorbid condition, previous joint surgery replacement or actual indication for joint surgery replacement, and inadequate health literacy. Unrealistic RP patients were classified as either optimistic patients (patients without significant RP but ≥7 criteria) or pessimistic patients (patients with significant RP but < 7 criteria).

### Sample size calculation

To detect an effect size of 20% (43% vs. 65% [12]) for the absolute difference in concordance with treatment between patients with and without significant RP, we estimated the sample size using a one-tailed test, a 5% significance level and a power of 85%. The G*Power estimate was a total sample size of 414 patients, 180 with significant RP and 234 without significant RP. The final sample and patient distribution obtained allowed us to have a power of 0.79.

### Statistical analysis

We performed a descriptive statistical analysis. Patients with significant RP were compared to their counterparts, as were patients with unrealistic RP vs. their counterparts. The Mann-Whitney *U* test was used to compare continuous variables when they did not show a normal distribution (Kolmogorov-Smirnov). Fisher’s exact test or *χ*^2^ test was used to compare proportions.

Stepwise forward multiple logistic regression analysis was used to investigate factors associated with significant RP and unrealistic RP. Variables included in the models tested were selected according to their statistical significance in the univariate analysis (*p* ≤ 0.05); previously, correlations between specific variables were analyzed, and when the Pearson correlation coefficient was ≥ 0.7, one of them was selected for inclusion in the model. In addition, age and sex were the only variables forced into the models as they have been related to disease severity. Finally, multiple logistic regression analysis was repeated when unrealistic RP definition was based on less/equal or more than 6 medical criteria.

There were no missing data. Additional statistical tests (to sample size estimation) were two-sided and evaluated at the 0.05 significance level. The statistical analysis was performed using the SPSS/PC program (v.17.0; Chicago IL).

## Results

### Population characteristics (Table 1)

During the study period, 450 consecutive RA outpatients were invited to participate; 35 of them declined. The characteristics of the 415 patients included are summarized in Table 1. Briefly, patients were primarily middle-aged females, with medium-low socioeconomic status, disease-specific autoantibodies, and substantial disease duration; patients had low levels of formal education and their RAPID-3 score indicated that a minority had severe disease activity. The patient-reported outcomes were generally favorable. Most of the patients had comorbidities. Almost half of the patients were receiving corticosteroids and a minority was receiving intensive RA-related treatment. Less than half of the patients were concordant with regard to treatment. The majority of the patients had health literacy. Finally, a positive family history of RA was recorded in almost half of the patients.

**Table 1:** Population characteristics and their comparison among patients with and without significant RP

Characteristics	Entire population, ***N*** = 415	Patients with significant RP, ***N*** = 94	Patients without significant RP, ***N*** = 321	***p***
**Socio-demographic**				
Female sex^1^	387 (93.3)	93 (98.9)	294 (91.6)	0.009
Years of age	55 (44.8–61.1)	57.8 (50–63)	54.3 (42–60)	0.001
Years of formal education	9 (6–13.3)	9 (6–12)	10 (6–14)	0.004
Medium-low SE level^1^	388 (93.5)	90 (95.7)	298 (92.8)	0.475
**RA-related**				
Serum positive RF^1^	373 (89.9)	83 (88.3)	290 (90.3)	0.562
Serum positive ACCP^1^	373 (89.9)	82 (87.2)	291 (90.7)	0.334
Years of disease duration	13.3 (8–18.6)	16.6 (12–20)	12.5 (7–18)	≤ 0.0001
Rheumatoid nodes^1^	95 (22.9)	49 (63.9)	46 (13.6)	≤ 0.0001
RAPID-3 score	4.7 (1.7–11.8)	13 (4–17)	3.2 (1.4–9)	≤ 0.0001
High disease activity category^1^	96 (23.1)	48 (51.1)	48 (15)	≤ 0.0001
**Patient-reported outcomes**				
Pain-VAS	5 (2–17.3)	14 (5.6–39)	4 (1–10)	≤ 0.0001
Overall disease-VAS	5.3 (1–20)	16 (5–39)	4 (1–16)	≤ 0.0001
HAQ score	0.25 (0–1.13)	1.13 (0.13–2)	0.13 (0–1)	≤ 0.0001
SF-36 score	71.9 (51.2–86.1)	54 (42–78.7)	75.5 (58–88)	≤ 0.0001
Mental component score (SF-36)	75 (54.6–88.2)	57 (46.8–81)	78 (59.4–90)	≤ 0.0001
Physical component score (SF-36)	68.9 (48.9–86.3)	47.8 (39–76)	75 (54–88.4)	≤ 0.0001
**Comorbidity**				
Presence of comorbidity^1^	255 (61.4)	72 (76.6)	183 (75)	0.001
Surgical joint replacement^1^	87 (21)	58 (61.7)	29 (9)	≤ 0.0001
**Treatment**				
DMARDs/patient	1 (1–2)	2 (1–2)	1 (1–2)	0.008
Corticosteroids use^1^	182 (43.9)	38 (40.4)	144 (44.9)	0.480
Intensive treatment^1^	66 (15.9)	19 (20.2)	47 (14.6)	0.202
**Compliance with treatment**				
Adherence^1^	227 (54.7)	46 (48.9)	182 (56.7)	0.196
Persistence^1^	228 (54.9)	41 (43.6)	186 (57.9)	0.018
Concordance^1^	183 (44.1)	33 (35.1)	150 (46.7)	0.058
**Miscellaneous**				
Health Literacy^1^	329 (79.3)	80 (85.1)	249 (77.6)	0.147
Adequate RA knowledge^1^ (based on CQ)	195 (47)	31 (33)	164 (51)	0.002
RA family history^1^	186 (44.8)	58 (61.7)	128 (39.9)	≤ 0.0001

Data are presented as the median (IQR) unless otherwise indicated

^1^Number (%) of patients

*SE* socioeconomic, *RF* rheumatoid factor, *ACCP* antibodies to cyclic citrullinated peptides, *RAPID-3* Routine Assessment of Patients Index Data-3 score, *VAS* visual analogue scale, *HAQ* Health Assessment Questionnaire, *SF-36* Short Form-36, *DMARDs* disease-modifying anti-rheumatic drugs, *CQ* Compliance Questionnaire, *RA* rheumatoid arthritis

### Factors associated with significant RP

There were 94 patients whose scores indicated significant RP (22.6%), and their characteristics were compared to the 321 patients (77.4%) without significant RP (Table 1**)**. Briefly, patients with significant RP were more frequently females, older, and with fewer years of formal education; they also had longer disease duration, more severe disease, and higher disease activity; accordingly, they also showed deteriorated patient-reported outcomes compared to their counterparts. Patients with significant RP also had more frequent comorbidity and indicated a more intensive treatment; meanwhile, a lower percentage of them were persistent on treatment. Finally, patients with significant RP more frequently reported a positive family history of RA and less frequently had RA-related knowledge.

In the regression analysis to identify factors associated with significant RP, the following variables were included: female sex, age, years of formal education (highly correlated to adequate RA knowledge that was not included in the model), years of disease duration, rheumatoid nodes and RAPID-3 score (highly correlated to high disease activity category, pain-VAS and overall disease-VAS, all were excluded), HAQ and SF-36 scores, surgical joint replacement (highly correlated to comorbidity that was excluded), number of DMARDs per patient, persistence on therapy (highly correlated with concordance with therapy that was excluded), and family history of RA. Table 2 summarizes the results; compliance with therapy (and related outcomes) was not retained in the model; meanwhile, rheumatoid nodes, surgical joint replacement, family history of RA and RAPID-3 score were all associated with significant RP (*R*^2^ = 0.455).

**Table 2:** Regression analysis to identify factors associated with significant RP

	OR	95% CI	***p***
Rheumatoid nodes	12.36	6.78–22.53	≤ 0.0001
Surgical joint replacement	2.14	1.12–4.09	0.022
RA family history	1.94	1.08–3.49	0.027
RAPID-3 score	1.12	1.07–1.12	≤ 0.0001

*OR* odds ratio, *CI* confidence interval, *RA* rheumatoid arthritis, *RAPID-3* Routine Assessment of Patients Index Data-3

### Unrealistic RP

**Fig. 1:**
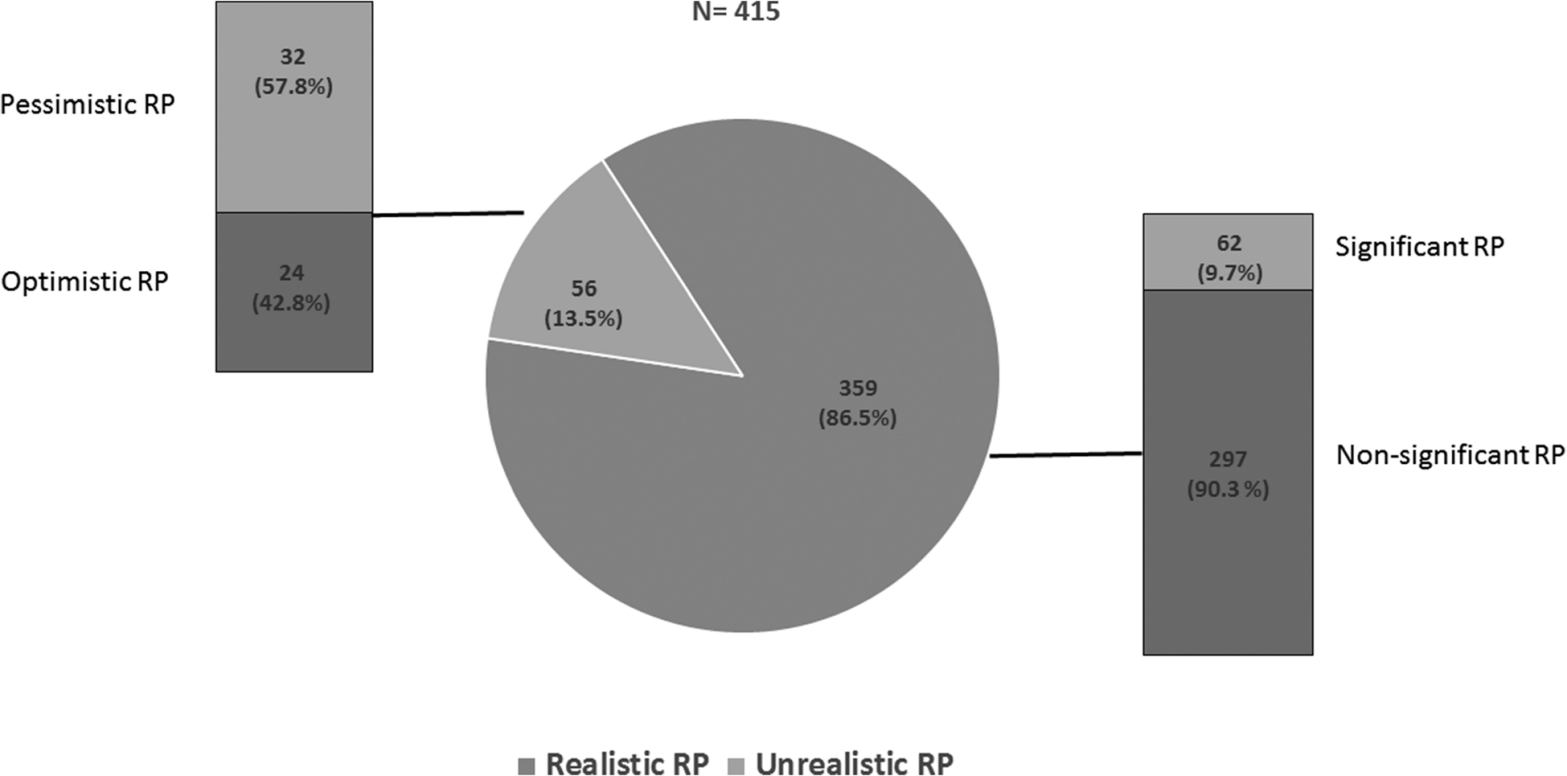
Patient distribution according to the presence/absence of significant RP and number of criteria

The 415 patients included were classified into four categories based on the presence/absence of significant RP and their coincidence with seven or more/less than seven unfavorable medical criteria, as summarized in Fig. 1. The majority of the patients were realistic, primarily without significant RP. Only 56 patients were unrealistic, among them 32 (57.1%) were pessimistic; there were expected differences when characteristics from both groups of unrealistic RP patients were compared, as summarized in Table 3**.**

**Table 3:** Comparison of the characteristics from unrealistic pessimistic RP patients and unrealistic optimistic RP patients

Characteristics	Unrealistic pessimistic RP patients, ***N*** = 32	Unrealistic optimistic RP patients, ***N*** = 24	***p***
**Socio-demographic**			
Female sex^1^	31 (96.9)	24 (100)	1
Years of age	59.5 (49.6–64.6)	57.1 (43.3–61.7)	0.329
Years of formal education	9 (6–15.8)	9 (6–12)	0.590
Medium-low SE level^1^	29 (90.6)	24 (100)	0.252
**RA-related**			
Serum positive RF^1^	24 (75)	24 (100)	0.008
Serum positive ACCP^1^	25 (78.1)	24 (100)	0.016
Years of disease duration	13.5 (8.7–18)	18.5 (7.4–25)	0.329
Rheumatoid nodes^1^	15 (46.9)	13 (54.2)	0.787
RAPID-3	4.1 (1.4–12.6)	15.3 (14.1–18.7)	0.002
High disease activity category^1^	6 (18.8)	19 (79.2)	≤ 0.0001
**Patient-reported outcomes**			
Pain-VAS	8 (3.3–14)	27.5 (7.6–36)	0.007
Overall disease-VAS	7.5 (2–15)	32.5 (7.5–45.8)	0.001
HAQ score	0.25 (0–1.1)	2 (1.4–2.5)	≤ 0.0001
SF-36 score	77.2 (55.4–87.3)	42.5 (22.6–67)	≤ 0.0001
Mental component score (SF-36)	71.9 (56.5–87.8)	48.1 (32–67)	0.002
Physical component score (SF-36)	74 (48.9–84.6)	39.7 (17.4–53.3)	≤ 0.0001
**Comorbidity**			
Presence of comorbidity^1^	19 (59.4)	19 (79.2)	0.153
Surgical joint replacement^1^	9 (28.1)	13 (54.2)	0.059
**Treatment**			
DMARDs/patient	1 (1–2)	2 (1–2)	0.165
Corticosteroids use^1^	11 (34.4)	18 (75)	0.003
Intensive treatment^1^	10 (31.3)	18 (75)	0.003
**Compliance with treatment**			
Adherence^1^	18 (56.3)	11 (45.8)	0.590
Persistence^1^	19 (59.4)	111 (45.8)	0.418
Concordance^1^	15 (46.9)	10 (41.7)	0.789
**Miscellaneous**			
Health Literacy^1^	24 (75)	22 (91.7)	0.162
Adequate RA knowledge^1^ (based on CQ)	25 (44.6)	170 (47.4)	0.774
RA family history^1^	18 (56.3)	10 (41.7)	0.418
**Significant RP**^**1**^	32 (100)	0	≤ 0.0001
**Unfavorable medical criteria**	5 (5–6)	7.5 (7–8)	≤ 0.0001

Data are presented as the median and IQR unless otherwise indicated

^1^Number (%) of patients

*SE* socioeconomic, *RF* rheumatoid factor, *ACCP* antibodies to cyclic citrullinated peptides, *RAPID-3* Routine Assessment of Patients Index Score-3, *VAS* visual analogue scale, *HAQ* Health Assessment Questionnaire, *SF-36* Short Form-36, *DMARDs* disease-modifying anti-rheumatic drugs, *CQ* Compliance Questionnaire, *RA* rheumatoid arthritis, *RP* risk perception

We then compared unrealistic patients to their counterparts, and the results are summarized in Table 4**.** Briefly, unrealistic patients tended to be older and had higher disease activity, worse patient-reported outcomes, more intensive treatment, and more frequent rheumatoid nodes and surgical joint replacement; they also had more frequent significant RP and a higher number of unfavorable medical criteria.

**Table 4:** Comparison of unrealistic and realistic RP patients’ characteristics

Characteristics	Unrealistic patients, ***N*** = 56	Realistic patients, ***N*** = 359	***p***
**Socio-demographic**			
Female sex^1^	55 (98.2)	332 (92.5)	0.152
Years of age	58.5 (46.4–63)	54.7 (44.8–60.7)	0.082
Years of formal education	9 (6–14.8)	9 (6–13)	0.372
Medium-low SE level^1^	53 (94.6)	335 (93.3)	1
**RA-related**			
Serum positive RF^1^	48 (85.7)	325 (90.5)	0.337
Serum positive ACCP^1^	49 (87.5)	324 (90.3)	0.481
Years of disease duration	14 (8.3–20.3)	13.2 (7.7–18.3)	0.224
Rheumatoid nodes^1^	28 (50)	67 (18.7)	≤ 0.0001
RAPID-3	10.8 (2–18)	4.2 (1.6–10)	0.004
High disease activity category^1^	25 (44.6)	71 (19.8)	≤ 0.0001
**Patient-reported outcomes**			
Pain-VAS	9.8 (5–31)	5 (2–16)	≤ 0.0001
Overall disease-VAS	10 (5–35)	5 (1–18)	0.001
HAQ score	1 (0–2.1)	0.25 (0–1.1)	0.001
SF-36 score	61.3 (41.1–81.1)	73 (54.3–87)	0.002
Mental component score (SF-36)	62.5 (42.9–86.9)	76.6 (56.9–88.4)	0.008
Physical component score (SF-36)	55.3 (38–80)	70.4 (51.4–87.8)	0.003
**Comorbidity**			
Presence of comorbidity^1^	38 (67.9)	217 (60.4)	0.306
Surgical joint replacement^1^	22 (39.3)	65 (18.1)	0.001
**Treatment**			
DMARDs/patient	1 (1–2)	1 (1–2)	0.031
Corticosteroids use^1^	29 (51.8)	153 (42.6)	0.247
Intensive treatment^1^	28 (50)	144 (40.1)	0.190
**Compliance with treatment**			
Adherence^1^	29 (51.8)	199 (55.4)	0.666
Persistence^1^	30 (53.6)	197 (54.9)	0.886
Concordance^1^	25 (44.6)	158 (44)	1
**Miscellaneous**			
Health Literacy^1^	46 (82.1)	283 (78.8)	0.723
Adequate RA knowledge^1^ (based on CQ)	25 (44.6)	170 (47.4)	0.774
RA family history^1^	28 (50)	158 (44)	0.470
**Significant RP**^**1**^	32 (57.1)	62 (17.3)	≤ 0.0001
**Unfavorable medical criteria**	6 (5–7)	5 (4–6)	≤ 0.0001

Data are presented as the median and IQR unless otherwise indicated

^1^Number (%) of patients

*SE* socioeconomic, *RF* rheumatoid factor, *ACCP* antibodies to cyclic citrullinated peptides, *RAPID-3* Routine Assessment of Patients Index Score-3, *VAS* visual analogue scale, *HAQ* Health Assessment Questionnaire, *SF-36* Short Form-36, *DMARDs* disease-modifying anti-rheumatic drugs, *CQ* Compliance Questionnaire, *RA* rheumatoid arthritis, *RP* risk perception

Finally, in the regression analysis to identify factors associated with unrealistic RP, the following variables were included: rheumatoid nodes, RAPID-3 score (highly correlated with pain-VAS and HAQ scores that were not included in the model), overall disease-VAS, SF-36 score, surgical joint replacement, number of DMARDs per patient, significant RP, and number of unfavorable medical criteria. The presence of significant RP [odds ratio (OR), 3.875; 95% confidence interval (CI), 1.875–8.010; *p* ≤ 0.0001] and number of unfavorable medical criteria (OR, 1.286; 95% CI, 1.032–1.602; *p* ≤ 0.0001) were independently associated with unrealistic RP (*R*^2^ = 0.177). Similar results were obtained when unrealistic RP definition was established with 6 unfavorable medical criteria (Additional file 4 Supplementary table); the number of medical criteria (OR, 2.215; 95% CI, 1.770–2.774; *p* ≤ 0.0001) and significant RP (OR, 1.190; 95% CI, 1.087–2.418; *p* ≤ 0.0001) were independently associated with unrealistic RP (*R*^2^ = 0.218).

## Discussion

Our primary hypothesis was that RA patients with significant RP would better adhere to DMARDs, which was based on the philosophical construct that doctor-patient relationships are guided by professionals’ concerns for the patient’s best interest [25]. There is also evidence that RA patients’ fears and beliefs about their disease may affect patients’ compliance with treatment; in a recent literature review [26], fears related to pharmacological therapy were the most frequently reported, and 20% of the articles included in the data analysis highlighted that patients’ beliefs about therapy affect adherence to treatment [27–29]. Meanwhile, fear of disability and about the future course of the disease (which could be included in the second and third dimensions, respectively, of our RPQ) had also been frequently cited as patient concerns [26, 30].

In the study, adherence, persistence, and concordance with therapy were lower among the patients with significant RP, although these variables were not maintained in the multivariate analysis. It is worth discussing these results that differ from those highlighted in the literature review [26]. First, the articles cited refer to RA patients’ fears/beliefs, and these terms, fears and risk, differ; “fear” is an emotion; meanwhile, “risk” is a cognitive process, the threat of quantifiable damage [31, 32]. In such context, our patients with significant RP may have presented with additional unsuspected psychological comorbidity such as depression and anxiety, which may have ultimately impacted adherence [29, 33]. Gossec et al. [30] developed the Fear Assessment in Inflammatory Rheumatic diseases (FAIR) questionnaire for fear assessment in French patients with RA or spondyloarthritis; in their national survey, almost one fifth of the patients evaluated had high fear scores, which were associated with psychological distress, although patients treatment behavior was not assessed. Interestingly, negative views about gout have also been associated with lower adherence to urate-lowering therapy [34]. Second, the methods used to assess patient fears/beliefs were diverse and included individual interviews and/or focus groups, generic [35–37] and disease-specific [30] questionnaires, which differ from our instrument in terms of their cultural background construction [12]. Last, there is a paucity of published articles reporting fears and beliefs in RA patients from Latin America. Nationality and ethnicity determine cultural background and influence patients’ perceptions about trust in their physicians, beliefs about RA-medications, and the choice of RA priority domains [26, 38–40]; all may shape the RP construct and lead to the lack of homogeneous results across countries. Moreover, rheumatic patients from the Latin America region have little access to private medicine and face widespread difficulty to obtain early specialized medical attention, which affects disease-related outcomes [41]. The COMOrbidities in RA (COMORA) multinational study [42] included RA patients from 17 countries; authors found that patients from countries with lower socioeconomic welfare scored worse on the majority of the physician-reported measures for disease activity; paradoxically, these patients evaluated their patient-global assessment and fatigue with similar or better scores, which was explained by lower income societies setting less pressure with regard to execution of social roles, and differences in patients expectations from the treatment, with patients from low-income countries having lower expectations and greater acceptance of achieved outcomes. Also, patients from our region expressed particular interests and concerns about their disease, such as myths and realities in eating habits and family involvement in RA care, as has been reported in a qualitative study that explored RA patients’ needs for educational material [43]; patient’s reliance of extended family for care was also proposed by Putrik et al. [42] as being more acceptable in RA patients from low-income countries compared to patients from wealthier but more individualist societies. Finally, high-power-distance cultural communities are characteristics of the Latin American region and determine a paternalistic patient-doctor relationship, which is not objected to by Mexican patients [3]. Ultimately, outcomes, patient’s perspective, and how patients interact with their rheumatologist are all contextual cultural factors that shape the RP construct [5].

We found that rheumatoid nodes, surgical joint replacement, positive family history of RA, and the RAPID-3 score were all associated with significant RP. Rheumatoid nodes, surgical joint replacement, and the RAPID-3 score may be considered to represent a more aggressive disease subset; accordingly, the association of these variables with significant RP seems intuitive. RA patients’ beliefs about their disease (a construct related to the RP construct) have been shown to impact different RA patients’ health domains, such as disability and quality of life [39]; in addition, illness perception, which is most commonly conceptualized as “the patient’s generic and organized cognitive representations or schemata that derive from prior experiences in the medical domain”, has been associated with worse outcomes in patients with chronic diseases, including 84 patients with RA [44]. Finally, in the study previously cited [30], French patients with high perceived disease activity were more frequently classified in the high fear cluster, after hierarchical cluster analysis was performed. Meanwhile, in a qualitative study where a similar population underwent an interview, hereditary and familial predisposition were cited by French RA patients (and patients with spondyloarthritis) as factors that shaped their beliefs about the origin of their rheumatologic disease [45]. A positive family history (in addition to age and female gender) has also been associated with the perceived risk of developing osteoporosis in Mexican men and women [46].

In the study, a minority of the patients (13.5%) had unrealistic RP, and the two variables independently associated were the presence of significant RP and a higher number of unfavorable medical criteria. Positive unrealistic thinking has been associated with inappropriate health behaviors, particularly (but not limited) to treatment non-adherence [17]. In the context of RA, there is published evidence indicating that patients have inappropriate perceptions and expectations about their disease and its treatment [45] and that there is a low concordance between (French) RA patient and physician perceptions of the impact of the disease on functioning [47]. Fournier et al. [48] examined the role of a three-dimensional approach to optimism in the adaptation to three chronic diseases, including RA; they found that positive efficacy expectancies were helpful when patients must deal with largely controllable diseases, where self-care is required, such as RA. Studies in patients with other health conditions (but RA) have identified different demographic, health-related, and behavioral characteristics associated with unrealistic optimism and pessimism [16]. Interestingly, in the study that characterized factors associated with judgment biases about breast cancer in 14,426 women, population segments that were already vulnerable to negative health outcomes displayed more unrealistic pessimism than less vulnerable populations [16]; we found a similar association between a higher number of unfavorable medical criteria (which could be a surrogate of negative health outcomes in the context of RA) and unrealistic RP. Finally, among patients with unrealistic RP, we found a slightly higher percentage of patients with unrealistic pessimism compared to those with unrealistic optimism; meanwhile, it has been described that people usually tend to underestimate their vulnerability and risk of health problems, which defines unrealistic optimism [49].

The study has some limitations that need to be addressed. First, this is a cross-sectional study and no causality can be inferred. In addition, the RP construct may vary as the disease progresses and we had a limited representation of patients with early disease. Second, significant RP was defined based on the 61.7-mm cut-off derived from the RPQ score distribution in the population where the questionnaire was validated. Third, potentially relevant variables such as beliefs about medications and trust in the physician were not assessed, and these have been associated with our relevant outcomes [28]. Fourth, unrealistic RP included patients with unrealistic optimism and pessimism, and the literature has shown different factors associated with each status [16]. Fifth, the number of unfavorable medical criteria to be considered for the unrealistic RP definition was arbitrarily selected. Finally, the study was performed in a particular population of Mexican RA patients, and their clinical, demographic, and ethnic characteristics may be relevant in shaping their RP construct and adherence to treatment, which limits the generalizability of the results.

## Conclusions

RP is a complex construct that can be considered a useful patient-reported outcome that evaluates patients’ perceived negative consequences of their disease in a quantitative manner. RA patients with significant RP and unrealistic RP show particular characteristics and could be considered a suitable target population for specific interventions aimed at improving the patient-physician dialog, which is required to bring the rheumatologists´ vision closer to the patient’s perception.

## Supplementary Information

**Additional file 1.** Comorbid conditions. List of comorbid conditions.

**Additional file 2.** The Compliance Questionnaire (CQ). The Compliance Questionnaire (CQ) Spanish and English versions.

**Additional file 3.** The Risk Perception Questionnaire (RPQ). The Risk Perception Questionnaire (RPQ) Spanish and English versions.

**Additional file 4.** Comparison of unrealistic and realistic patient characteristics, considering ≥6 unfavorable medical criteria. Comparison table of unrealistic and realistic patient characteristics, considering ≥6 unfavorable medical criteria.

## Data Availability

All data that support our findings are contained within the manuscript. Requests for further details on the dataset and queries related to data sharing arrangements may be submitted to the corresponding author.

## Abbreviations

RP: Risk perception
RA: Rheumatoid arthritis
RAPID-3: Routine Assessment of Patient Index Data-3
HAQ-DI: Health Assessment Questionnaire Disability Index
SF-36: Short Form-36
VAS: Visual analogue scale
DMARDs: Disease-modifying anti-rheumatic drugs
RPQ: Risk perception questionnaire
INCMyN-SZ: Instituto Nacional de Ciencias Médicas y Nutrición Salvador Zubirán
CQ: Compliance Questionnaire
RF: Rheumatoid factor
ACCP: Antibodies to cyclic citrullinated peptides
OR: Odds ratio
CI: Confidence interval
