# Supplementary material for: Markers of disease severity and positive family history are associated to significant risk perception in rheumatoid arthritis, while compliance with therapy is not: a cross-sectional study in 415 Mexican outpatients
Source: Arthritis Res Ther. 2021 Feb 22;23:61. doi: 10.1186/s13075-021-02440-y (PMC7898444; doi:10.1186/s13075-021-02440-y)
Supplement: Supplementary file 1 — Additional file 1. Comorbid conditions. List of comorbid conditions. [file 13075_2021_2440_MOESM1_ESM.pdf]

Additional file 1. List of comorbid conditions

Dyslipidemia	Epilepsy
Breast cancer	Liver disease
Biliary cirrhosis	Autoimmune hepatitis
Osteoporosis	Osteoporosis
Diabetes Mellitus	Anxiety disorder
Hypothyroidism	Vitiligo
Arterial hypertension	Pulmonary fibrosis
Fibromyalgia	Secondary Sjögren syndrome
Bipolar disorder	Venous insufficiency

Obesity was not considered a comorbid condition if affected patients were not under any medical intervention (exercise, diet, pharmacological), surgical intervention, and/or were not attending the obesity outpatient clinic.
