# Supplementary material for: Markers of disease severity and positive family history are associated to significant risk perception in rheumatoid arthritis, while compliance with therapy is not: a cross-sectional study in 415 Mexican outpatients
Source: Arthritis Res Ther. 2021 Feb 22;23:61. doi: 10.1186/s13075-021-02440-y (PMC7898444; doi:10.1186/s13075-021-02440-y)
Supplement: Supplementary file 2 — Additional file 2. The Compliance Questionnaire (CQ). The Compliance Questionnaire (CQ) Spanish and English versions. [file 13075_2021_2440_MOESM2_ESM.pdf]

INSTITUTO NACIONAL DE CIENCIAS MEDICAS Y NUTRICION SALVADOR ZUBIRAN

DEPARTAMENTO DE INMUNOLOGIA Y REUMATOLOGIA

AUTOEVALUACION DEL APEGO A TRATAMIENTO FARMACOLOGICO Y FACTORES ASOCIADOS A FALTA DE APEGO

Estimado paciente:

Los tratamientos médicos para controlar padecimientos como la Artritis Temprana son muy largos y es común que los pacientes olviden o dejen de tomar los medicamentos y falten a sus consultas, lo cual puede provocar que los resultados del tratamiento no sean los que se esperaban. Por este motivo estamos interesados en conocer cuales pudieran ser las razones que ayudarían a que los medicamentos no dejen de tomarse y así mejorar la atención médica que se le brinda.

La participación en este estudio es voluntaria y puede usted retirarla cuando así lo decida sin que esto interfiera en la atención que recibe en el Instituto.

Lo invitamos a colaborar con nosotros contestando el siguiente cuestionario

Fecha de la entrevista: |__|__| |__|__| |__|__|
Día Mes Año

Nombre: ____
Apellido Paterno Apellido Materno Nombre (s)

No. de registro: |__|__|__|__|__|__|

1 Ocupación Actual |__|

1	Ama de casa	4	Empleo informal	7	Otro
2	Estudiante	5	Desempleado		
3	Empleo formal	6	Pensionado		

2 Nivel socioeconómico |__|

1	3	5
2	4	6

3 ¿Además del medicamento indicado por su médico tomo algún otro tipo de tratamiento alternativo, durante los últimos 3 meses?

|__| 1 Si 2 No

En caso de respuesta positiva ¿Cuál? ______________________

____

4 En los últimos 3 meses dejó de tomar el medicamento indicado por su médico, para consumir algún otro tipo de producto indicado por algún tratamiento alternativo?

4 Siempre
3 Casi siempre
2 Algunas Veces
1 Casi nunca
0 Nunca

5 Por favor califique que tanta confianza le tiene a su reumatólogo tratante.
Si considera que 0 es nada de confianza y 10 toda
la confianza

6 Por favor califique que tan bien ha entendido las indicaciones que le ha dado su reumatólogo tratante para tomar sus medicamentos. Si considera que 0 es nada bien y 10 Muy bien

7 a) Por favor califique la calidad de las consultas que ha recibido de su reumatólogo en los últimos 3 meses, si considera que 0 es Ninguna calidad y 10 La máxima calidad.

b) Por favor califique la calidad de las consultas de laboratorio que ha recibido en los últimos 3 meses, si considera que 0 es Ninguna calidad y 10 La máxima calidad.

8 ¿En los últimos 3 meses ¿qué tanta dificultad tuvo para encontrar sus medicamentos para la artritis en la farmacia?

4 Demasiada
3 Mucha
2 Más o menos
1 Un poco
0 Nada

9 ¿En los últimos 3 meses ¿qué tan caros le parecieron los medicamentos para la artritis que le indicó su médico?

4 Demasiado
3 Mucho
2 Más o menos
1 Un poco
0 Nada

10 ¿En los últimos 3 meses ¿qué tan seguido ha dejado de tomar sus medicamentos?

4 Siempre
3 Casi siempre
2 Algunas Veces
1 Casi nunca
0 Nunca

*** Si en la pregunta anterior contestó que dejó de tomar sus medicamentos para la artritis casi nunca, algunas veces, casi siempre ó siempre, por favor conteste la siguiente pregunta.**
Si contestó la opción nunca, pase a la pregunta 12 en la hoja siguiente.

11 Por favor, lea los siguientes enunciados y responda cruzando con una x a cada una de las posibles razones por las cuales dejo de tomar sus medicamentos en los últimos 3 meses

11.1	Porque no tuve dinero para comprarlo	☐ Si	☐ No
11.2	Porque no lo encontré en la farmacia	☐ Si	☐ No
11.3	Porque no me hace sentir mejor	☐ Si	☐ No
11.4	Porque me siento más mal si me lo tomo	☐ Si	☐ No
11.5	Porque el medicamento es muy caro	☐ Si	☐ No
11.6	Porque se me olvida tomarlo	☐ Si	☐ No
11.7	Porque si no me lo tomo, no pasa nada	☐ Si	☐ No
11.8	Porque son muchos fármacos los que tengo que tomar	☐ Si	☐ No
11.9	Porque tuve que hacer más cosas de las que hago normalmente en el día	☐ Si	☐ No
11.10	Porque hice menos cosas de las que acostumbro siempre	☐ Si	☐ No
11.11	Porque nadie me lo recordó	☐ Si	☐ No
11.12	Porque la hora en que tomo mis medicamentos no coincide con la hora de mis alimentos	☐ Si	☐ No
11.13	Porque no me encontraba en casa a la hora de tomarlo	☐ Si	☐ No
11.14	Porque no lo había comprado	☐ Si	☐ No
11.15	Porque salí de viaje	☐ Si	☐ No

* Si desea anotar alguna otra razón puede hacerlo en la línea siguiente

____

12 En los últimos 3 meses he tomado mis medicamentos el día exacto en que me lo ha indicado mi médico ☐

4 Siempre
3 Casi siempre
2 Algunas Veces
1 Casi nunca
0 Nunca

13 En los últimos 3 meses he tomado mis medicamentos en el horario en que me lo ha indicado mi médico ☐

4 Siempre
3 Casi siempre
2 Algunas Veces
1 Casi nunca
0 Nunca

14 En los últimos 3 meses cada vez que he tomado mis medicamentos, he tomado el número total de tabletas que me ha indicado mi reumatólogo ☐

4 Siempre
3 Casi siempre
2 Algunas Veces

1 Casi nunca
0 Nunca

15 Considera usted que la Artritis reumatoide es una enfermedad:

a) Crónica ☐ b) Pasajera ☐ ☐ No sé

16 En caso de tener necesidad de hablar con alguien ¿cuenta usted con algún confidente que pueda escucharlo?

☐ Si ☐ No

17 ¿Considera usted que la Artritis Reumatoide es una enfermedad que se puede curar?

☐ Si ☐ No ☐ No sé

18 En caso de tener necesidad económica ¿cuenta usted con alguien que le preste dinero?

☐ Si ☐ No

19 ¿Considera usted que la Artritis Reumatoide es una enfermedad hereditaria?

☐ Si ☐ No ☐ No sé

20 Si usted requiere de saber algo con respecto a su salud, ¿cuenta con alguien a quien siente la confianza de preguntarle sobre la información que desee saber?

☐ Si ☐ No

21 ¿Usted cree que alguien que padece Artritis Reumatoide debe hacer ejercicio?

☐ Si ☐ No ☐ No sé

22 Si desea hablar o ver a algún familiar, ¿cuenta con alguno cercano para que pueda hacerlo?

☐ Si ☐ No

MUCHAS GRACIAS POR HABER CONTESTADO ESTE CUESTIONARIO

Compliance Questionnaire

Dear patient:

Medical treatments that help to control symptoms from diseases like yours are frequently indicated for a long period of time. Sometimes, patients forget or stop taking their medications, or missed a medical appointment what may account in lesser therapy effectiveness than previously expected.

We are interested in knowing possible reasons which may help you to continue taking your medication as prescribed in order to improve your medical attention.

Your participation in this study is voluntary. You may stop participating whenever you decide and if so, it will not interfere with the existing medical attention at the Institution.

You are invited to collaborate by answering the following survey.

Interview date: Day, Month, Year

Name: First Last name, Second Last name, Name(s)

Institution identification number:

1 .- Actual occupation

1	Housewife	4	Non-officially employed	6	Retired
2	Student	5	Unemployed	7	Other
3	Officially employed				

2.- Socioeconomic classification at the Institution

1	90% gratuity	3	70% gratuity	5	50% gratuity
2	80% gratuity	4	60% gratuity	6	40% gratuity

3.- Have you taken any alternative therapy, additionally to the treatment prescribed by the rheumatologist in charge of your care?

1 Yes 2 No If the answer is yes please specified which one

4.- During the past 3 months, did you stop taking the medication prescribed by your rheumatologist because of any reason including the choice of alternative medicine?

4. Always 3. Almost always 2. Sometimes 1.Almost never 0. Never

5.- Please rate in a scale from 0 to 10, how much you trust your rheumatologist.

0 indicates no trust at all and 10 indicates all the possible trust.

6.- Please rate in a scale from 0 to 10, how well you have understood treatment indications given by the rheumatologist in charge of your care.

0 indicates no understanding of medical indications regarding treatment and 10 indicates a perfect understanding.

7a.- Please rate in a scale from 0 to 10 the quality of the rheumatic evaluations you received. 0 indicates the poorest quality and number 10 the best quality.

7b.- Please rate in a scale from 0 to 10 the quality of central laboratory appointments you received.

0 indicates the poorest quality and number 10 the best quality (excellence).

8.- In the past 3 months, how difficult it was to find your arthritis medications at the pharmacy?

4. Too much   3. Much.   2. Some   1. A little   0. Any

9.- In the past 3 months, how expensive were the medications for arthritis that your rheumatologist indicated you?
4. Too much   3. Much.   2. Some   1. A little   0. Any

10.- In the past 3 months, how often did you completely stop taking your medication?
4. Always   3. Almost always. 2. Sometimes   1. Almost never   0. Never

**If you have answered numbers 4 (always), 3 (almost always), 2 (sometimes) or 1 (almost never), please answer the following question as well (question number 11).*

**If you have answered number 0 (never), please go to question number 12*

11.- Please read the following sentences and cross with an X each sentence you consider it was a reason to stop taking your medication during the past 3 months. *You may choose more than one answer*

11.1- Because I had no money	Yes	No
11.2- Because it was not available at the drugstore	Yes	No
11.3- Because it does not make me feel better	Yes	No
11.4- Because it may me feel worse when I take it	Yes	No
11.5- Because the medication is very expensive	Yes	No
11.6- Because I forget to take it	Yes	No
11.7- Because nothing happens if I do not take it	Yes	No
11.8- Because I am taking a lot of medication at this time	Yes	No
11.9- Because I had to do more things than I usually do through the day	Yes	No
11.10- Because I did fewer things than I usually do through the day	Yes	No
11.11- Because nobody reminded me to take my medication	Yes	No
11.12- Because timing/s when my medication is prescribed is different from my mealtime/s	Yes	No
11.13- Because I was not at home when I had to take my medication	Yes	No
11.14- Because I did not buy it	Yes	No
11.15- Because I went out on a trip	Yes	No

***** If you wish to write some other reason/s, you may do it in the following space………………………………………………………………………

12.- In the past 3 months, I took my medication exactly at the day/s indicated by my rheumatologist
4. Always   3. Almost always. 2. Sometimes   1. Almost never   0. Never

13.- In the past 3 months, I took my medication exactly at the day-times indicated by my rheumatologist
4. Always   3. Almost always. 2. Sometimes   1. Almost never   0. Never

14.- In the past 3 months, every time I took my medication, I took the precise amount of tablets indicated by my rheumatologist
4. Always   3. Almost always. 2. Sometimes   1. Almost never   0. Never

15.- You consider that Rheumatoid Arthritis is ….
a) A chronic disease   b) A disease that will resolve   c) I do not know

16.- Do you have any confident to talk with? Yes No

17.- Do you consider that Rheumatoid Arthritis is a curable disease?
Yes No I do not know

18.- If you have an economical urgency is there somebody who can help you? Yes No

19.- Do you consider that Rheumatoid arthritis is an inherited disease?
Yes No I don't know

20.- If you have doubts about your health, is there somebody trustworthy to talk with? Yes No

21.- Do you believe that someone who has rheumatoid arthritis should exercise?
Yes No I don't know

22.- Do you have relatives to talk or spend time with them? Yes No

THANK YOU VERY MUCH FOR HAVING ANSWERED THIS QUESTIONNAIRE

Items 1 and 2 are related to demography; items 3 and 4 are related to the use of alternative medicine (yes/no and modality); items 5 and 6 evaluate patient-physician relationship; in item 7 patients qualify the quality of physician's evaluation and central laboratory facilities; in item 8, patients use a Likert scale (0 to 4) to determine his perception about the supply of rheumatologic medicine; in item 9, patients use a Likert scale (0 to 4) to determine his perception about the costs of the rheumatologic medicine; in item 10, patients use a Likert scale (0 to 4) to determine persistence on therapy; item 11 investigates patients reasons of inadequate medication taking behavior and includes 15 predefined answers (most of them obtained from literature review) and one open answer; in items 12 to 14, patients use a Likert scale to evaluate adherence to DMARD therapy; items 15, 17, 19 and 21 investigate patient's knowledge about the disease (scored from 0 if no answer is correct to 4 if all the items are correctly answered); finally, items 16, 18, 20 y 22 determine the level of social support (scored from 0 to 4, if all the items are answered as Yes).
