# Supplementary material for: Markers of disease severity and positive family history are associated to significant risk perception in rheumatoid arthritis, while compliance with therapy is not: a cross-sectional study in 415 Mexican outpatients
Source: Arthritis Res Ther. 2021 Feb 22;23:61. doi: 10.1186/s13075-021-02440-y (PMC7898444; doi:10.1186/s13075-021-02440-y)
Supplement: Supplementary file 4 — Additional file 4. Comparison of unrealistic and realistic patient characteristics, considering ≥6 unfavorable medical criteria. Comparison table of unrealistic and realistic patient characteristics, considering ≥6 unfavorable medical criteria. [file 13075_2021_2440_MOESM4_ESM.pdf]

Supplementary table. Comparison of unrealistic and realistic patient characteristics, considering ≥ 6 unfavorable medical criteria.

Characteristics	Unrealistic patients, N=86	Realistic patients, N=329	p
**Socio-demographic**			
Female sex[1]	85 (98.8)	302 (91.8)	0.016
Years of age	58.5 (46-64.2)	54.6 (44.1-60.5)	0.005
Years of formal education	9 (6-12.5)	9 (8-14)	0.061
Medium-low SE level[1]	84 (97.7)	304 (92.4)	0.088
**RA-related**			
Serum positive RF[1]	79 (91.9)	294 (89.4)	0.688
Serum positive ACCP[1]	81 (94.2)	292 (88.8)	0.162
Years of disease duration	14.1 (8.3-23.2)	13 (7.7-18)	0.019
Rheumatoid nodes[1]	30 (34.9)	65 (19.8)	0.004
RAPID-3	9.3 (2.2-15.7)	4 (1.4-9.7)	≤0.0001
High disease activity category[1]	38 (44.2)	58 (17.6)	≤0.0001
**Patients-reported outcomes**			
Pain-VAS	9.3 (4-33)	5 (2-14)	≤0.0001
Overall disease-VAS	11 (4-35)	5 (1-17)	≤0.0001
HAQ score	1 (0.9-1.88)	0.125 (0-1)	≤0.0001

SF-36 score	60.6 (46.8-80.1)	74.3 (54.6-87.7)	≤0.0001
Mental component score (SF-36)	63.3 (50.5-83.9)	78.1 (56.7-88.8)	≤0.0001
Physical component score (SF-36)	55.45 (40.1-76.2)	73.8 (52.6-88.5)	≤0.0001
**Comorbidity**			
Presence of comorbidity[1]	67 (77.9)	188 (57.1)	≤0.0001
Surgical joint replacement[1]	23 (26.7)	64 (19.5)	0.14
**Treatment**			
DMARDs/patient	1 (1-2)	1 (1-2)	≤0.0001
Corticosteroids use[1]	51 (59.3)	131 (39.8)	0.001
Intensive treatment[1]	26 (30.2)	40 (12.2)	0.000
**Compliance with treatment**			
Adherence[1]	39 (45.3)	189 (57.4)	0.052
Persistence[1]	67 (77.9)	256 (77.8)	1
Concordance[1]	32 (37.2)	151 (45.9)	0.18
**Miscellaneous**			
Health Literacy[1]	74 (86)	255 (77.5)	0.1
Adequate RA knowledge[1] (based on CQ)	37 (43)	158 (48)	0.467
RA family history[1]	35 (40.7)	151 (45.9)	0.559
**Significant RP[1]**	35 (40.7)	74 (22.5)	0.001
**Unfavorable medical criteria**	6 (6-7)	5 (4-5)	≤0.0001

Data are presented as the median and IQR unless otherwise indicated. [1]Number (%) of patients. SE=socioeconomic. RF=rheumatoid factor. ACCP=antibodies to cyclic citrullinated peptides. RAPID-3=Routine Assessment of Patients Index Score-3. VAS=visual analogue scale. HAQ=Health Assessment Questionnaire. SF-36=Short Form-36. DMARDs=disease modifying anti-rheumatic drugs. CQ=Compliance Questionnaire. RA= rheumatoid arthritis. RP=risk perception.
